# Racial and Ethnic Disparities in the Prescribing of Pain Medication in US Primary Care Settings, 1999–2019: Where Are We Now?

**DOI:** 10.1007/s11606-024-08638-5

**Published:** 2024-02-01

**Authors:** Trevor Thompson, Sofia Stathi, Jae Il Shin, Andre Carvalho, Marco Solmi, Chih-Sung Liang

**Affiliations:** 1 Centre for Chronic Illness and Ageing, University of Greenwich, London, UK; 2 Centre for Inequalities, University of Greenwich, London, UK; 3 Department of Pediatrics, Yonsei University College of Medicine, Seoul, Republic of Korea; 4 Innovation in Mental and Physical Health and Clinical Treatment (IMPACT) Strategic Research Centre, School of Medicine, Barwon Health, Deakin University, Geelong, VIC Australia; 5 Department of Psychiatry, University of Ottawa, Ottawa, ON Canada; 6 Ottawa Hospital Research Institute (OHRI) Clinical Epidemiology Program University of Ottawa, Ottawa, ON Canada; 7 Department of Child and Adolescent Psychiatry, Charité Universitätsmedizin, Berlin, Germany; 8 Department of Psychiatry, Beitou Branch, Tri-Service General Hospital, Taipei, Taiwan

**Keywords:** analgesia, pain, NAMCS, disparities, race, ethnicity, primary care

## Abstract

**Background:**

Policy initiatives have attempted to reduce healthcare inequalities in the USA, but evidence on whether these initiatives have reduced racial and ethnic disparities in pain treatment in primary care is lacking.

**Objective:**

To determine whether racial and ethnic disparities in medication prescribed for pain in primary care settings have diminished over a 21-year period from 1999 to 2019.

**Design:**

An annual, representative cross-sectional probability sample of visits to US primary care physicians, taken from the National Ambulatory Medical Care Survey.

**Patients:**

Pain-related visits to primary care physicians.

**Main Measures:**

Prescriptions for opioid and non-opioid analgesics.

**Key Results:**

Of 599,293 (16%) sampled visits, 94,422 were pain-related, representing a population-weighted estimate of 143 million visits made annually to primary care physicians for pain. Relative risk analysis controlling for insurance, pain type, and other potential confounds showed no difference in pain medication prescribed between Black and White patients (*p* = .121). However, White patients were 1.61 (95% CI 1.32–1.97) and Black patients 1.57 (95% CI 1.26–1.95) times more likely to be prescribed opioids than a more underrepresented group consisting of Asian, Native-Hawaiian/Pacific-Islander, and American-Indian/Alaska-Natives (*p*s < .001). Non-Hispanic/Latino patients were 1.32 (95% CI 1.18–1.45) times more likely to receive opioids for pain than Hispanic/Latino patients (*p* < .001). Penalized cubic spline regression found no substantive narrowing of disparities over time.

**Conclusions:**

These findings suggest that additional intervention strategies, or better implementation of existing strategies, are needed to eliminate ethnic and racial disparities in pain treatment towards the goal of equitable healthcare.

**Supplementary Information:**

The online version contains supplementary material available at 10.1007/s11606-024-08638-5.

## INTRODUCTION

A landmark Institute of Medicine (IOM) report commissioned by the US Congress over two decades ago documented inequities in healthcare^1^ and highlighted racial and ethnic disparities in pain treatment.^2^ Such disparities have been consistently demonstrated in the emergency department (ED) with Black, Asian, and Hispanic patients markedly less likely to receive opioid medication than White and non-Hispanic patients.^3^ Disparities in pain medication prescribed in primary care settings have also been examined, with such settings differing to the ED in immediacy of access, consultation time, perceived care quality and proportion of chronic pain patients.^1^ Black and Hispanic patients are also less likely than White patients to have a primary care provider and more commonly use the ED for pain treatment.^4,5^ Several national studies of primary care settings have found Hispanic patients to be less likely to be prescribed opioid analgesics^6–10^ or to receive lower dosages^11^ typically even after controlling for potential confounds such as insurance status, pain condition, and region. Similar findings have been reported for Black patients in some^7,9^ but not other^8,11^ studies, with limited research on racially underrepresented groups such as Asian, Native-Hawaiian/Pacific-Islander (NHPI), and American-Indian/Alaska-Natives (AIAN).

Despite broad political and regulatory initiatives^12^ for promoting equitable healthcare, such as the Affordable Care Act,^13^ it is unclear to what extent these have impacted inequities across racial and ethnic groups in pain care. One study^7^ of US national primary care data found that the lower rate of opioid prescriptions for back and abdominal pain in Hispanic and Black compared to non-Hispanic White patients was similar across data averaged within 2006–2010 and 2011–2015 time periods. However, there is no clear picture on the extent to which disparities have systematically changed over time since the commissioning of the IOM report on health inequalities in 1999^1^ and which includes the period from 2016 when significant changes were made to opioid prescribing guidelines.^14^

The current study examines racial and ethnic disparities in pain medication prescribed in US primary care settings across a 21-year period from 1999 to the latest available patient data provided by the Centers for Disease Control in 2019. Based on previous findings, we hypothesize that White patients are more frequently prescribed opioids for pain than Black patients or an underrepresented group consisting of Asian, Native-Hawaiian/Pacific-Islander (NHPI), and American-Indian/Alaska-Natives (AIAN), and non-Hispanic/Latino patients are more frequently prescribed opioids than Hispanic/Latino patients. While disparities are expected to vary over the study period, no predictions are made on the direction of changes given a lack of previous data.

## METHOD

### Study Design and Setting

This study examines patient record data from the National Ambulatory Medical Care Survey (NAMCS) and is reported in accordance with STROBE guidelines (Appendix A). The NAMCS is an annual probability sample of patient visits to non-federally employed office-based physicians in the USA administered by the National Center for Health Statistics (NCHS). A detailed description of the sampling methods is available elsewhere.^15^ In brief, a multistage probability design is used, with ~ 1300 physicians randomly sampled from within geographical strata. The physician and/or supporting staff record data from ~ 30 randomly selected patient visits over a 1-week reporting period, which includes patient demographics, medications prescribed, and reasons for visit. Patients’ reasons for visit are recorded verbatim and subsequently reclassified by medical coders who assign up to three reason-for-visit classes using the NCHS standardized classification system.^15^ Coding accuracy is subject to independent quality control procedures and error rates are typically < 1%.^15^

### Study Sample

Records for pain-related visits were identified from NAMCS datasets spanning 1999–2019, except for 2017 which is not yet released due to COVID-related processing delays. A visit was categorized as pain-related if the patient’s main reason-for-visit code matched any of the classification codes^15^ relating to pain (Appendix B). Data were combined across different annual survey waves as described in Appendix C.

### Race and Ethnicity

Race and ethnicity are abstracted directly from patient medical records in the NAMCS and categorized based on National Institute of Health reporting standards. For ethnicity, two categories are used: Hispanic/Latino and not Hispanic/Latino. For race, five categories are used: White, Black/African-American, Asian, Native-Hawaiian/Pacific-Islander (NHPI), and American-Indian/Alaska-Native (AIAN). A NAMCS-recoded three-category variable of White, Black, and Other Race (Asian, NHPI, AIAN) is provided for analysis given prohibitively small sample sizes for NHPI and AIAN subgroups and is employed in the current study. Appendix D gives full definitions of race and ethnicity categories.

As missing data are high for race and ethnicity (25.5% and 22.7% in the current data), NAMCS provides imputed variables for each survey year (except for ethnicity in 1999–2002 surveys). Imputation involves a model-based sequential regression using key predictors described in detail elsewhere.^15^

### Outcomes

Two binary outcomes were used: whether opioids were prescribed (yes/no), and (ii) whether non-opioid analgesics only were prescribed (yes/no). Opioids were indicated if any prescribed medication had a Multum^16^ class of 575860 (narcotic analgesics) or 5758191 (narcotic analgesic combinations). Non-opioid analgesics only were indicated if any medications matched classes 575861 (NSAIDs), 575862 (salicylates), or 5758278 (cox-2 inhibitors) and none matched an opioid class. If a patient’s medication matched class 575863 (general analgesic combinations), ingredient codes were examined to determine medication type.

### Model Covariates

To assess the independent association of race and ethnicity with medication outcomes, we included several covariates commonly previously examined in studies of racial and ethnic disparities and that may influence opioid prescribing.^17^ These included patient sex, age, insurance (private/Medicaid/Medicare/none/unknown), pain chronicity (acute/chronic/pre- or post-surgery/preventive), and new patient status (new/existing). We also controlled for alcohol and substance misuse/dependence (Appendix E) as these may influence physicians’ willingness to prescribe opioids. In addition, we controlled for pain type, after recoding 53 identified pain codes into eight broader classes of musculoskeletal, abdominal, chest (excluding heart), headache, eye/ear, dental, genitourinary, and other pain (Appendix E). Practice characteristics controlled for were census region (Northeast/Midwest/South/West) and metropolitan status (metropolitan/non-metropolitan). Finally, we included survey year and age as first-degree (linear) and second-degree (quadratic) orthogonal polynomials based on previously observed relationships.^18^

### Statistical Analysis

We examined racial and ethnic differences in prescribed medication using log-link Poisson regression to estimate both crude relative risks (RRs), and adjusted RRs after including all covariates. Race and ethnicity were dummy coded^19^ with White and Hispanic/Latino used as reference groups. To obtain a direct comparison of the non-reference groups for race (Black and Other Race), we reran the analysis after re-referencing to Other Race.

To determine whether the magnitude of race disparities changed across survey years in a linear or quadratic fashion, we added first then second-degree Race × Date interaction terms and sequentially assessed change in model fit with likelihood ratio tests. This procedure was repeated for ethnicity. To explore any possible more complex non-linear trends, we also performed regression using natural cubic splines,^20^ fitting a series of smoothed local polynomial regressions across equally spaced time intervals. If this suggested an obvious pattern of higher-order changes in disparities across time, we reran initial models adding the appropriate polynomial interactions.

Analyses were performed with the survey package^21^ in R using NAMCS-provided patient weights to compute nationally representative estimates and sampling unit design variables to adjust for clustering. Multiple visits by the same patient are not identifiable in NAMCS data, but the 1-week recording period means repeated visits should be relatively uncommon and introduce little additional non-independence.

In accordance with NCHS guidelines,^15^ statistical significance was set to *α* = 0.01 to reduce type I error rate.

### Sensitivity Analysis

Although NAMCS imputation of missing race and ethnicity data would seem unlikely to introduce large systematic bias, we nevertheless examined alternative imputations. We applied the principles of threshold analysis^22^ to examine what the true pattern in the missing/imputed data would need to be to nullify group differences, so that reasonable judgements can be made about whether such patterns in the missing data are plausible.

For ethnicity, we iteratively replaced NAMCS-imputed ethnicity data (*N* = 23,182) within the whole dataset (*N* = 94,421) with simulated data representing a range of relative risks. We then reran the main analysis on each whole dataset and from these analyses, identified the least extreme relative risk in the simulated/missing data that resulted in ethnicity becoming non-significant. The same procedure was performed for race. Given that relative risks for Black and White (vs. Other Race) were similar in the main analysis, relative risks for these comparisons were constrained to be equal in each simulation to reduce computational time.

## RESULTS

### Demographic and Clinical Characteristics of Pain-Related Visits

Of 599,293 NAMCS patient records, 94,422 were classified as pain-related, representing a population-weighted estimate of over 143 million annual primary care visits for pain (eTable 1), or 16% of all physician visits. Musculoskeletal, “other” (primarily post-operative), and abdominal pain were the most common types of pain for all racial and ethnic groups (eFigure 1). Racial and ethnic composition of visits was White (85%), Black (10%), Other Race (5%), and non-Hispanic/Latino (88%), Hispanic/Latino (12%).

Visits characteristics are shown in Table 1, with statistically significant differences (*p* < 0.01) within racial and ethnic groups observed for all characteristics except substance and alcohol misuse/dependence. Differences were generally small, with type of pain (eFigure 1), sex, chronicity, and new patient status showing a similar distribution for each group. More marked dissimilarities were evident for insurance, age, and geographic region. Black and Hispanic patients were twice as likely, and Other Race around 1.5 times as likely, to be Medicaid beneficiaries compared to White and non-Hispanic/Latino patients. Black and Hispanic/Latino patients also had a notably lower representation of ≥ 65-year-old adults compared to all other groups.

**Table 1:** Population-Weighted Distribution (%) of Patient and Practice Characteristics Recorded for Pain-Related Visits to US Office-Based Physicians 1999–2019 (exc. 2017) in Total Sample of 94,422 Visit Records

		Race			Ethnicity			
Characteristic		White	Black	Other	Hispanic/Latino	Non-Hispanic/Latino	Sample size^*^	Estimated visits in population^†^
Race	White	100.0	0.0	0.0	92.7	84.3	81,462	2,442,908,518
	Black	0.0	100.0	0.0	4.6	10.7	9015	285,438,728
	Other	0.0	0.0	100.0	2.7	5.0	3945	134,890,293
Ethnicity	Hispanic/Latino	13.3	5.7	7.1	100.0	0.0	10,071	351,409,646
	Non-Hispanic/Latino	86.7	94.3	92.9	0.0	100.0	84,351	2,511,827,893
Sex	Female	59.6	64.3	61.9	61.7	60.0	55,887	1,724,076,615
	Male	40.4	35.7	38.1	38.3	40.0	38,535	1,139,160,923
Age	0–17 years	11.1	10.2	10.6	15.8	10.3	9729	313,447,269
	18–29 years	8.5	9.7	7.9	10.6	8.4	8210	247,205,184
	30–64 years	53.3	59.8	54.3	53.4	54.1	51,888	1,546,785,161
	65 + years	27.1	20.4	27.2	20.2	27.3	24,595	755,799,925
Payment	Private insurance	56.4	50.6	53.1	50.4	56.4	51,271	1,593,752,417
	Medicaid	8.1	16.2	11.9	16.7	8.0	8859	260,672,824
	Medicare	24.4	21.0	21.4	17.6	24.8	22,933	684,167,453
	No insurance	5.1	5.1	6.6	7.3	4.9	4802	148,594,373
	Unknown	6.0	7.1	7.0	8.0	5.9	6557	176,050,473
Alcohol	No	98.2	98.5	98.3	98.5	98.2	92,683	2,812,558,488
	Yes	1.8	1.5	1.7	1.5	1.8	1739	50,679,051
Substance	No	99.4	99.5	99.1	99.6	99.4	93,834	2,846,350,998
	Yes	0.6	0.5	0.9	0.4	0.6	588	16,886,541
Pain chronicity	Acute	45.6	45.9	48.6	52.2	44.9	38,195	1,285,596,057
	Chronic (> 3 months)	34.8	36.4	36.7	31.0	35.6	33,798	984,453,544
	Pre-/post-surgery	17.4	14.5	11.6	13.9	17.3	18,861	473,601,241
	Preventive care	2.2	3.2	3.1	2.9	2.2	1954	64,523,754
Pain class	Abdominal	8.2	9.1	11.1	12.8	7.8	6806	240,688,253
	Musculoskeletal	47.1	47.9	48.3	43.0	47.8	40,626	1,352,962,449
	Other	23.8	19.6	17.4	19.2	23.6	25,896	659,502,739
	Eye/ear	7.1	5.2	6.2	7.6	6.8	5948	196,919,986
	Headache	5.4	6.3	6.2	6.9	5.4	6373	159,181,667
	Chest	3.6	6.1	4.4	4.6	3.8	3631	112,495,220
	Genitourinary	4.4	5.4	5.9	5.6	4.5	4802	131,360,899
	Dental	0.3	0.4	0.6	0.3	0.4	340	10,126,326
Patient status	Existing patient	83.6	83.1	83.8	82.6	83.7	76,515	2,393,358,800
	New patient	16.2	16.7	16.0	17.2	16.1	17,778	465,732,698
Region^‡^	Northeast	20.5	19.2	15.5	19.0	20.3	16,187	528,595,490
	Midwest	22.0	16.6	9.5	8.7	22.6	22,975	547,982,469
	South	35.3	52.5	19.4	37.7	36.1	31,191	952,384,918
	West	22.1	11.8	55.6	34.7	21.0	21,784	594,193,816
Rurality^‡^	Metropolitan area	86.8	91.4	96.6	95.2	86.7	70,695	2,367,128,965
	Non-Metropolitan	13.2	8.6	3.4	4.8	13.3	10,945	331,101,449

^*^The number of patient visits sampled across 1999–2019 (excluding 2017)

^†^The estimated number of actual visits across 1999–2019 (excluding 2017) in the US population

^‡^Data not collected for region (2018–2019) or rurality (2012)

### Opioid Medication

Population-weighted relative risks of medication receipt are shown in Table 2. The probability of receiving an opioid prescription for pain was 1.32 [95% CI 1.19–1.47] times greater for non-Hispanic/Latino (14.8%, 95% CI 14.1–15.6%) compared to Hispanic/Latino (11.2%, 95% CI 10.0–12.4%) patients (*p* < 0.001). Black patients were 1.60 [95% CI 1.33–1.92] times as likely and White patients 1.48 [95% CI 1.27–1.74] times as likely to be prescribed opioids than those from the Other Race group (*p* < 0.001). Overall probability of opioid receipt was 15.6% [95% CI 14.2–17.1%] for Black, 14.5% [95% CI 12.7–15.2%] for White, and 9.7% [95% CI 8.3–11.4%] for Other Race. There were no differences between Black and White patients (RR = 1.08, 95% CI 0.98–1.19, *p* = 0.121). Adjusted RRs for both ethnicity and race did not change substantively after controlling for covariates (Table 2).

**Table 2:** Population-Weighted Relative Risk [95% Confidence Intervals] of Receiving Medication During Pain-Related Visits to Office-Based Physicians (*N* = 77,748)

			Opioid medication		Non-opioid analgesics	
	Group	Reference	Relative risk^*^	*p*	Relative risk	*p*
Race	Black	White^†^	0.97 [0.89 1.07]	.579	1.14 [1.06, 1.22]	< .001
	Other		0.62 [0.51, 0.76]	< .001	1.03 [0.83, 1.27]	.783
Ethnicity	Not Hispanic or Latino	Hispanic/Latino	1.32 [1.18, 1.45]	< .001	0.79 [0.74, 0.86]	< .001
Sex	Male	Female	1.07 [1.01, 1.13]	.024	1.04 [0.99, 1.09]	.099
Payment	Medicaid	Private insurance	1.41 [1.28, 1.54]	< .001	1.01 [0.92, 1.11]	.801
	Medicare		1.38 [1.27, 1.50]	< .001	0.97 [0.90, 1.04]	.336
	No insurance		1.30 [1.11, 1.51]	.001	0.85 [0.74, 0.99]	.033
	Unknown		1.16 [1.04, 1.30]	.010	0.95 [0.85, 1.06]	.352
Alcohol disorder	Disorder	No disorder	1.37 [1.20, 1.56]	< .001	1.09 [0.92, 1.29]	.336
Substance disorder	Disorder	No disorder	2.21 [1.74, 2.80]	< .001	0.95 [0.66, 1.38]	.801
Chronicity	Chronic	Acute	1.35 [1.26, 1.44]	< .001	0.73 [0.69, 0.77]	< .001
	Pre-/post-surgery		0.82 [0.74, 0.92]	.001	0.53 [0.47, 0.59]	< .001
	Preventative care		0.63 [0.37, 1.07]	.087	0.71 [0.56, 0.90]	.004
Pain class	Musculoskeletal	Abdominal	2.21 [1.90, 2.57]	< .001	2.63 [2.29, 3.03]	< .001
	Other		1.63 [1.40, 1.91]	< .001	1.55 [1.33, 1.81]	< .001
	Eye/ear		0.67 [0.53, 0.84]	.001	0.96 [0.81, 1.13]	.601
	Headache		1.48 [1.23, 1.77]	< .001	2.17 [1.86, 2.54]	< .001
	Chest		0.92 [0.71, 1.18]	.504	2.62 [2.25, 3.05]	< .001
	Genitourinary		0.72 [0.56, 0.92]	.009	1.01 [0.84, 1.22]	.909
	Dental		5.44 [4.01, 7.38]	< .001	1.68 [1.05, 2.69]	.031
New patient	New patient	Existing patient	0.74 [0.67, 0.80]	< .001	0.93 [0.87, 0.98]	.011
Region	Midwest	Northeast	1.19 [1.04, 1.36]	.011	1.05 [0.94, 1.16]	.384
	South		1.36 [1.20, 1.55]	< .001	0.98 [0.90, 1.07]	.643
	West		1.36 [1.19, 1.55]	< .001	0.99 [0.89, 1.11]	.921
Metropolitan area	Urban	Rural	0.92 [0.80, 1.05]	.217	0.98 [0.90, 1.07]	.670
Age	Linear		1.17 [1.11, 1.22]	< .001	1.13 [1.09, 1.17]	< .001
	Quadratic		0.60 [0.57, 0.63]	< .001	0.99 [0.96, 1.02]	.662
Survey year	Linear		1.22 [1.17, 1.27]	< .001	1.03 [0.99, 1.07]	.121
	Quadratic		0.96 [0.92, 1.01]	.106	1.06 [1.02, 1.10]	.002
	(Intercept)		0.03 [0.02, 0.04]	< .001	0.11 [0.09, 0.14]	< .001

^*^Relative risk > 1 indicates a greater probability of receiving a prescription compared to the reference group

^†^Reparamaterisation of the model with Black as the reference group to produce a direct comparison of Black vs. Other Race, gave RR = 1.57 [1.26, 1.95], *p* < .001 for opioid medication and RR = 1.10 [0.89, 1.37], *p* = .380 for non-opioid medication

Table 2 also indicates increased opioid prescribing for existing patients, chronic pain patients, certain pain classes, and South and West institutions. Private health insurance was associated with reduced opioid prescribing. A quadratic association of age was also found (eFigure 2), with a peak probability of opioid receipt occurring at around 50 years old.

**Figure 1:**
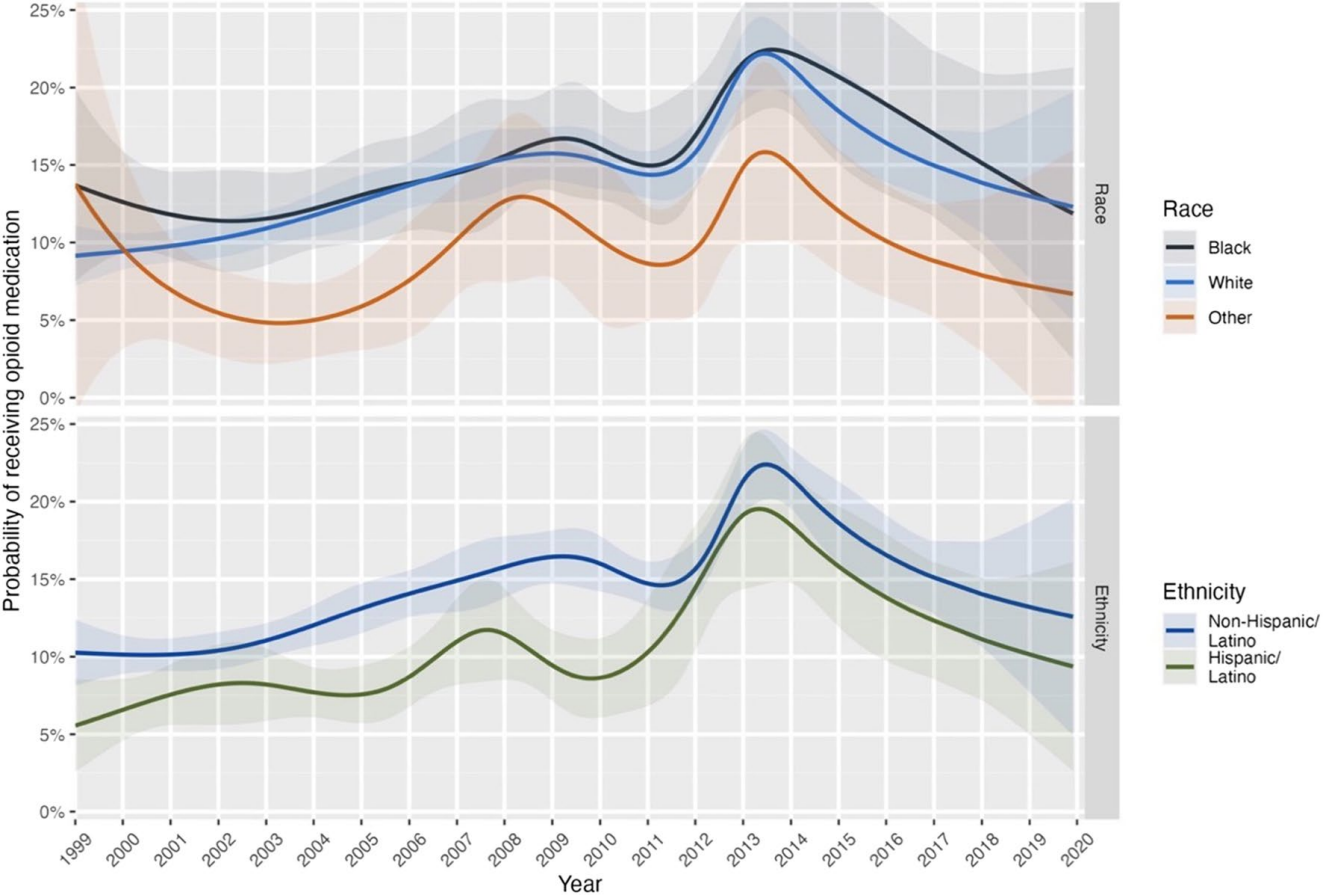
Smoothed probability of receiving a prescription for opioid medication during a visit to an office-based physician for pain.

With regard to overall prescribing trends, opioid prescriptions increased from around 10% in 1999 to a peak of 21% in 2013–2014 followed by a rapid decline. However, Fig. 1 (also eTable 2) suggests no substantive change in the magnitude of racial disparities, with the Other Race group showing a consistently lower probability of opioid receipt throughout 1999–2019. This was supported by polynomial regression which found no linear (*p* = 0.62) or quadratic (*p* = 0.26) changes in racial disparities across time. Hispanic/Latino patients also showed a consistently lower probability of opioid receipt. While Fig. 1 suggests this discrepancy might narrow slightly from around 2011, no significant linear (*p* = 0.062) or quadratic (*p* = 0.081) changes were found.

Opioids most commonly prescribed for all groups were acetaminophen-hydrocodone, tramadol, and acetaminophen-oxycodone (eFigure 3).

### Non-opioid Analgesics

The probability of being prescribed non-opioid analgesics only was 1.21 [95% CI 1.11–1.32, *p* < 0.001] times higher for Hispanic/Latino (21.6%, 95% CI 19.8–22.6%) compared to non-Hispanic/Latino (17.8%, 95% CI 17.2–18.5%) patients. Relative risks did not diminish after controlling for covariates (RR = 1.26 [95% CI 1.16–1.36], *p* < 0.001). There were no significant differences across Black vs. Other Race (*p* = 0.48) or White vs. Other Race (*p* = 0.33), but the proportion of non-opioid analgesic-only prescriptions was significantly higher for Black compared to White patients (RR = 1.15 [95% CI 1.07–1.24], *p* < 0.001). Table 2 also shows chronic pain, certain pain classes, and older age (eFigure 2) were associated with an increased probability of receiving only non-opioid analgesics.

**Figure 2:**
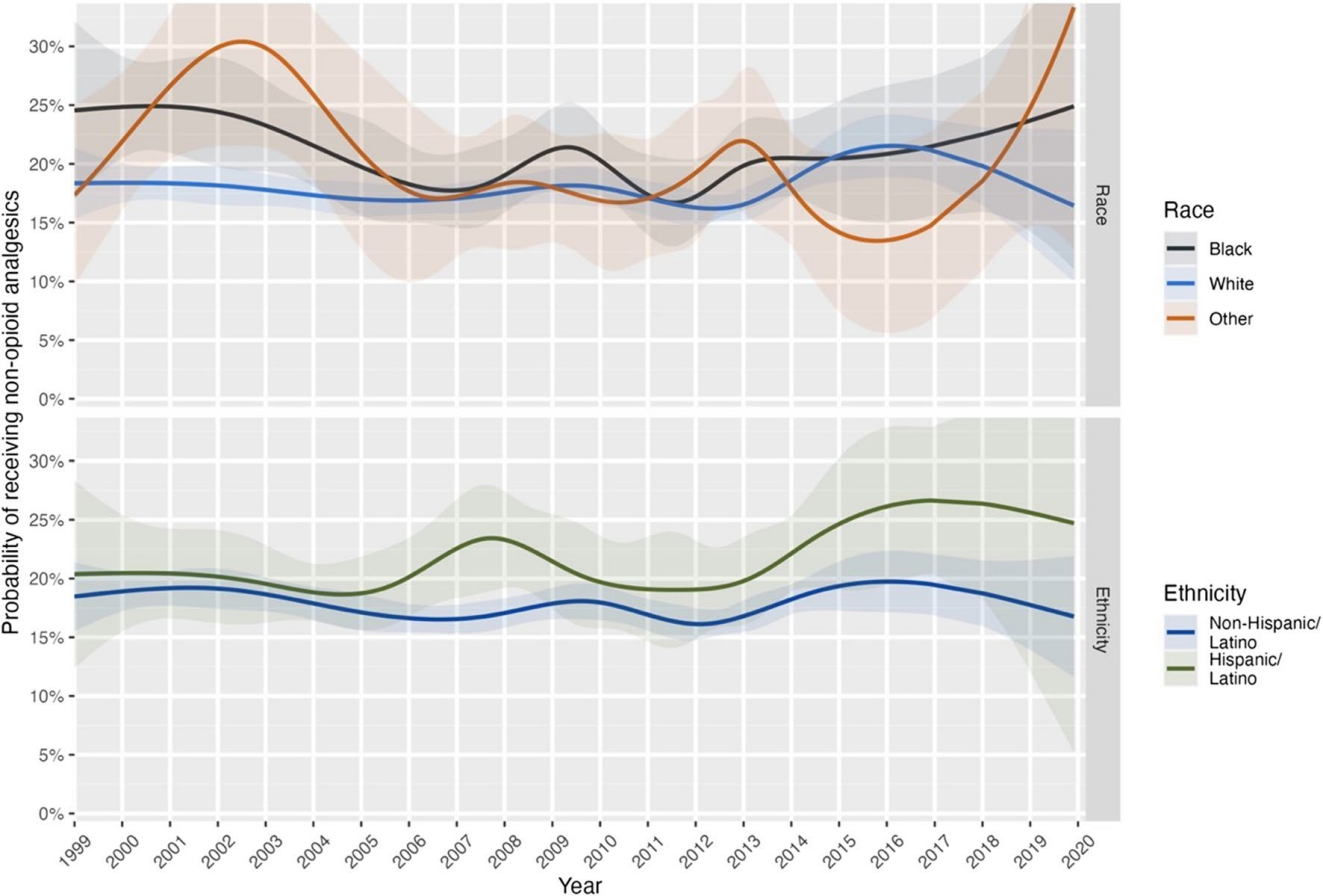
Smoothed probability of receiving a prescription for non-opioid analgesics only during a visit to an office-based physician for pain.

Figure 2 (also eTable 3) shows a relatively constant rate overall of non-opioid analgesic prescribing until a marked increase around 2012–2013. Although there is some suggestion that this increase is evident primarily for Hispanic/Latino patients, no significant linear (*p* = 0.297) or quadratic (*p* = 0.789) effects were found. There were also no linear (*p* = 0.066) or quadratic (*p* = 0.617) effects for race. The most common analgesics provided when no opioids were prescribed were Ibuprofen, Aspirin, and Naproxen (eFigure 4).

### Sensitivity Analysis

Threshold analysis of NAMCS-imputed data found that, for overall race differences in opioid prescribing to become non-significant, the missing Other Race group would have to have been at least 1.6 and 1.3 times as likely as Black and White patients respectively to receive an opioid prescription. For ethnicity differences to be nullified, missing Hispanic/Latino patients would have to be at least 1.3 times as likely as non-Hispanic/Latinos to receive an opioid prescription. These findings therefore indicate that a strong reversal of direction would have to be present in the missing/imputed data to nullify the significant race and ethnicity differences in the main analyses. We also conducted analysis after excluding NAMCS-imputed data (*N* = 66,726) and found the same pattern of results, albeit with RRs slightly higher compared to the imputed dataset for Black vs. Other Race (1.95 v 1.60) and White v Other Race (1.80 v 1.48), but with no other differences in RRs.

## DISCUSSION

Analysis of 94,422 records of patient visits to US office-based physicians for pain found significant racial and ethnic disparities in prescriptions for opioid medication, with little evidence of any narrowing of these disparities across the 21-year period from 1999 to 2019. Non-Hispanic/Latino patients were 1.32 times as likely overall to be prescribed opioids and 0.79 times as likely to be prescribed only non-opioid analgesics compared to Hispanic/Latinos, after controlling for insurance status, pain type, age, and other variables. White and Black patients were respectively 1.61 and 1.57 times as likely to receive opioid medication for pain compared to the Other Race group comprised of Asian, Native-Hawaiian/Pacific-Islanders (NHPI), and American-Indian/Alaska-Natives (AIAN) populations. Although these underrepresented subgroups are combined as a single group in NAMCS data due to limited NHPI and AIAN group sizes, census data indicates a likely composition of around 80% Asian, 17% AIAN, and 3% NHPI.^23^ No differences were found between White and Black patients, contrary to most studies of ED settings where White patients receive more opioid prescriptions.^3,24,25^ Although the reasons for this apparent discrepancy across ED and primary care settings are unclear, differences in time pressures, pain severity, chronicity, and consultation time^13^ are likely to be influential.^13^

To our best knowledge, this is the first investigation of pain medication prescribed in primary care that compares the more underrepresented racial groups with White and Black patient populations in a broad representative sample, and corroborates recent findings examining older Medicare patients seeking treatment for new low back pain.^9^ The lack of previous research is likely to be attributable to the difficulty of achieving sufficiently large samples, but is nevertheless surprising given that the Institute of Medicine report noted a particular need for further research on Asian, NHPI, and AIAN populations.^1^ Although visits to office-based physicians for pain by these patients collectively represent only 5% of all pain-related visits, this still constitutes around 7 million visits annually (Table 1). Furthermore, census projections indicate Hispanic and Asian populations to be the fastest growing subgroups in the US with population estimates for Asian Americans alone projected to almost triple to 62 million by 2065.^26^ Therefore, working to eliminate disparities in these groups is fundamental to future efforts for improving the nation’s health.

It is important to note that the choice of the “best” medication is dependent upon a considered clinical judgement of what is most appropriate for the presenting condition, and disparities in opioid prescribing do not automatically equate to inferior pain treatment. Nevertheless, the existence of substantive racial and ethnic disparities, especially after controlling for major potential confounds, does not seem to be easily explainable by differences in clinical presentation. Although the current data present robust evidence of the continued existence of disparities in pain care, they do not allow the determination of underlying mechanisms. Several explanations can nevertheless be considered. One, a significant proportion of the US Hispanic and Asian population report speaking English less than “very well” impairing their ability to communicate their health symptoms.^1,12^ Combined with time pressures, this can lead to diagnostic uncertainty and a reluctance to prescribe potentially inappropriate opioid medication. Two, physicians’ unconscious biases, including beliefs that minority groups are more easily addicted^25^ or have lower pain sensitivity^27^ despite evidence to the contrary^12,28^ may affect prescribing. Notably, however, unconscious biases have typically been previously investigated for Black and White patients, and we found no differences in prescribing between these two groups. Three, Asians and AIANs may be less likely to express pain due to embedded cultural values of stoicism or unwillingness to ask for help^12^ and Hispanic patients may reject opioids due to fears over addiction and side effects.^29^ Four, patients from specific underrepresented populations may exhibit greater reluctance to accept physician treatment recommendations based on a legacy of mistrust of the medical community and thus may be more likely to reject opioid treatment.^1^

The lack of narrowing of ethnic and racial disparities across 1999–2019 suggests legal, regulatory, and policy efforts^30–32^ to eliminate healthcare disparities have been unsuccessful with regard to pain treatment. Key recommendations of the Institute of Medicine report^1^ commissioned by Congress in 1999 included improving access to healthcare for underserved groups, better cross-cultural education during medical training to correct unconscious biases, and improving provider-patient communication by greater recruitment of underrepresented workers into healthcare. While legislation such as the Affordable Care Act has undoubtedly improved access to care for underserved groups,^33^ a failure to sufficiently implement the latter objectives may underlie the apparent continued existence of disparities in actual treatment.

Several limitations should be noted. To minimize administrative burden, NAMCS records minimal patient-provider encounter details and so offers limited insight into why disparities occur. In addition, unmeasured potential confounds such as reduced opioid availability in poorer, predominantly Black neighborhoods^25,34^ that might influence prescribing would not be adequately captured by the census region variable. We were, nevertheless, able to adjust for several important variables, and findings would be unlikely to be reversed with additional covariates. It is important to note that factors such as insurance status could plausibly occupy mediating, explanatory roles by, for example, acting as a class signaller which could affect providers’ empathic reactions and consequently their prescribing behavior. If this is the case, treating such variables as confounds by controlling for them could potentially result in an underestimation of RRs, and future studies should employ causal analysis models to help examine alternative model specifications.

A further limitation is the high proportion of imputed race and ethnicity data; although the general pattern of disparities observed is consistent with other studies^6–8,10^ and threshold analysis suggested that disparities would disappear only under relatively implausible conditions. NAMCS also does not assess pain severity; although previous evidence has typically found underrepresented groups to report greater, not less, pain^12,35^ compared to non-Hispanic or White patients. Medication and diagnostic information are also provider-reported so may not be optimally reliable, although verification checks have generally been favorable.^15^ Finally, while the aggregation of Asian, NHPI, and AIAN patients into a single group due to small samples is useful in broadly indicating the primary care experiences of the most underrepresented populations, it is impossible to determine whether this masks differential care experiences amongst these subgroups.

In summary, an analysis of 94,422 patient records observed no differences between Black and White patients in medication prescribed for pain but found that other underrepresented races and Hispanic/Latino patients were markedly less likely to receive prescriptions for opioid medication. There was little evidence for change in these prescribing disparities across a 21-year period from 1999 to 2019. Overall, these findings suggest that despite political and regulatory efforts, additional intervention strategies or better implementation of existing strategies are needed to eliminate disparities and achieve the goal of equitable healthcare.

### Supplementary Information

Below is the link to the electronic supplementary material.

Supplementary file1 (DOCX 255 KB)

Supplementary file2 (DOCX 33 KB)

## Data Availability

This study uses publicly available data which can be downloaded from the CDC website (https://www.cdc.gov/nchs/ahcd/datasets_documentation_related.htm).
