# Supplementary material for: Racial and Ethnic Disparities in the Prescribing of Pain Medication in US Primary Care Settings, 1999–2019: Where Are We Now?
Source: J Gen Intern Med. 2024 Feb 1;39(9):1597–605. doi: 10.1007/s11606-024-08638-5 (PMC11254888; doi:10.1007/s11606-024-08638-5)
Supplement: Supplementary file 1 — Supplementary file1 (DOCX 255 KB) [file 11606_2024_8638_MOESM1_ESM.docx]

**SUPPLEMENTARY MATERIAL**

[Supplementary Tables 2](#_Toc140145275)

[eTable 1. Number of pain-related visits each year to U.S. office-based physicians recorded in the NAMCS survey and estimated to have occurred in the population. 2](#_Toc140145276)

[eTable 2. Percentage of pain-related visits by race and ethnicity where an opioid prescription was issued. 3](#_Toc140145277)

[eTable 3. Percentage of pain-related visits by race and ethnicity where non-opioid medication only was prescribed. 4](#_Toc140145278)

[Supplementary Figures 5](#_Toc140145279)

[eFigure 1. Population-weighted frequency of pain classifications across race and ethnicity for pain-related visits from NAMCS data 1999-2019. 5](#_Toc140145280)

[eFigure 2. Population-weighted probability of medication receipt across age for pain-related visits. Second-order polynomial (quadratic) terms were used for model fitting. Points represent observed data, indicating the proportion of patients that receiving medication within one-year intervals. 6](#_Toc140145281)

[eFigure 3. Most common opioid medications provided for pain-related visits. 7](#_Toc140145282)

[eFigure 4. Most common non-opioid analgesics for pain-related visits when no opioids provided. 8](#_Toc140145283)

[Appendices 9](#_Toc140145284)

[Appendix A. STROBE Checklist. 9](#_Toc140145285)

[Appendix B. Classification of pain-related visits 11](#_Toc140145286)

[Appendix C. Combining annual data across survey years 12](#_Toc140145287)

[Appendix D. NAMCS patient record form instructions for coding ethnicity and race 13](#_Toc140145288)

[Appendix E. ICD-CM classification codes for alcohol or substance abuse/dependence 14](#_Toc140145289)

[References 15](#_Toc140145290)

# Supplementary Tables

## eTable 1. Number of pain-related visits each year to U.S. office-based physicians recorded in the NAMCS survey and estimated to have occurred in the population.

| **Survey Year** | **NAMCS  Sampled Visits** | **Estimated Visits in Population** |
| --- | --- | --- |
| 1999 | 3,012 | 104,785,321 |
| 2000 | 3,753 | 112,594,734 |
| 2001 | 3,291 | 111,089,040 |
| 2002 | 4,081 | 121,480,520 |
| 2003 | 4,237 | 156,212,832 |
| 2004 | 4,573 | 158,405,644 |
| 2005 | 4,371 | 163,284,441 |
| 2006 | 4,696 | 153,823,853 |
| 2007 | 5,172 | 166,159,358 |
| 2008 | 4,894 | 160,262,971 |
| 2009 | 5,469 | 176,801,447 |
| 2010 | 4,961 | 172,394,968 |
| 2011 | 4,811 | 151,368,361 |
| 2012 | 12,782 | 165,007,125 |
| 2013 | 9,382 | 157,638,572 |
| 2014 | 6,893 | 129,516,925 |
| 2015 | 4,086 | 152,591,144 |
| 2016 | 1,673 | 109,739,437 |
| 2017* | - | - |
| 2018 | 1,275 | 116,777,407 |
| 2019 | 1,010 | 123,303,439 |
| Annual Average | 4,721 | 143,161,877 |
| Total | 94,422 | 2,863,237,539 |

*NAMCS data not yet available.

## eTable 2. Percentage of pain-related visits by race and ethnicity where an opioid prescription was issued.

|  | **RACE** | | | **ETHNICITY** | |
| --- | --- | --- | --- | --- | --- |
| Year | Black | White | Other | Hispanic | Non-Hispanic |
| 1999 | 13% | 9% | 14% | 10% | 8% |
| 2000 | 12% | 10% | 5% | 11% | 4% |
| 2001 | 12% | 9% | 8% | 10% | 9% |
| 2002 | 8% | 11% | 3% | 11% | 9% |
| 2003 | 16% | 11% | 5% | 12% | 8% |
| 2004 | 12% | 11% | 8% | 11% | 9% |
| 2005 | 15% | 14% | 4% | 14% | 7% |
| 2006 | 12% | 14% | 13% | 15% | 9% |
| 2007 | 16% | 16% | 8% | 16% | 13% |
| 2008 | 17% | 14% | 15% | 15% | 8% |
| 2009 | 13% | 16% | 16% | 16% | 9% |
| 2010 | 16% | 15% | 5% | 15% | 11% |
| 2011 | 17% | 15% | 12% | 16% | 12% |
| 2012 | 19% | 18% | 9% | 18% | 14% |
| 2013 | 24% | 21% | 15% | 21% | 21% |
| 2014 | 21% | 24% | 13% | 24% | 21% |
| 2015 | 16% | 16% | 13% | 17% | 9% |
| 2016 | 21% | 15% | 1% | 14% | 17% |
| 2017* | - | - | - | - | - |
| 2018 | 17% | 12% | 9% | 13% | 9% |
| 2019 | 12% | 14% | 7% | 14% | 11% |

*NAMCS data not yet available.

## eTable 3. Percentage of pain-related visits by race and ethnicity where non-opioid medication only was prescribed.

|  | **RACE** | | | **ETHNICITY** | |
| --- | --- | --- | --- | --- | --- |
| Year | Black | White | Other | Hispanic | Non-Hispanic |
| 1999 | 21% | 18% | 18% | 18% | 19% |
| 2000 | 30% | 18% | 28% | %19% | 23% |
| 2001 | 20% | 19% | 25% | 20% | 18% |
| 2002 | 30% | 17% | 36% | 19% | 22% |
| 2003 | 18% | 18% | 29% | 19% | 18% |
| 2004 | 21% | 16% | 15% | 17% | 16% |
| 2005 | 20% | 18% | 24% | 18% | 21% |
| 2006 | 18% | 16% | 20% | 16% | 22% |
| 2007 | 19% | 18% | 18% | 17% | 23% |
| 2008 | 19% | 18% | 16% | 17% | 23% |
| 2009 | 22% | 18% | 16% | 18% | 19% |
| 2010 | 21% | 17% | 20% | 17% | 20% |
| 2011 | 15% | 18% | 20% | 18% | 22% |
| 2012 | 18% | 14% | 15% | 15% | 15% |
| 2013 | 19% | 18% | 21% | 18% | 22% |
| 2014 | 23% | 19% | 24% | 19% | 22% |
| 2015 | 20% | 22% | 11% | 19% | 25% |
| 2016 | 23% | 23% | 26% | 22% | 30% |
| 2017* | - | - | - | - | - |
| 2018 | 19% | 16% | 10% | 15% | 19% |
| 2019 | 26% | 19% | 35% | 19% | 32% |

*NAMCS data not yet available.

# Supplementary Figures

## eFigure 1. Population-weighted frequency of pain classifications across race and ethnicity for pain-related visits from NAMCS data 1999-2019.

## eFigure 2. Population-weighted probability of medication receipt across age for pain-related visits. Second-order polynomial (quadratic) terms were used for model fitting. Points represent observed data, indicating the proportion of patients that receiving medication within one-year intervals.


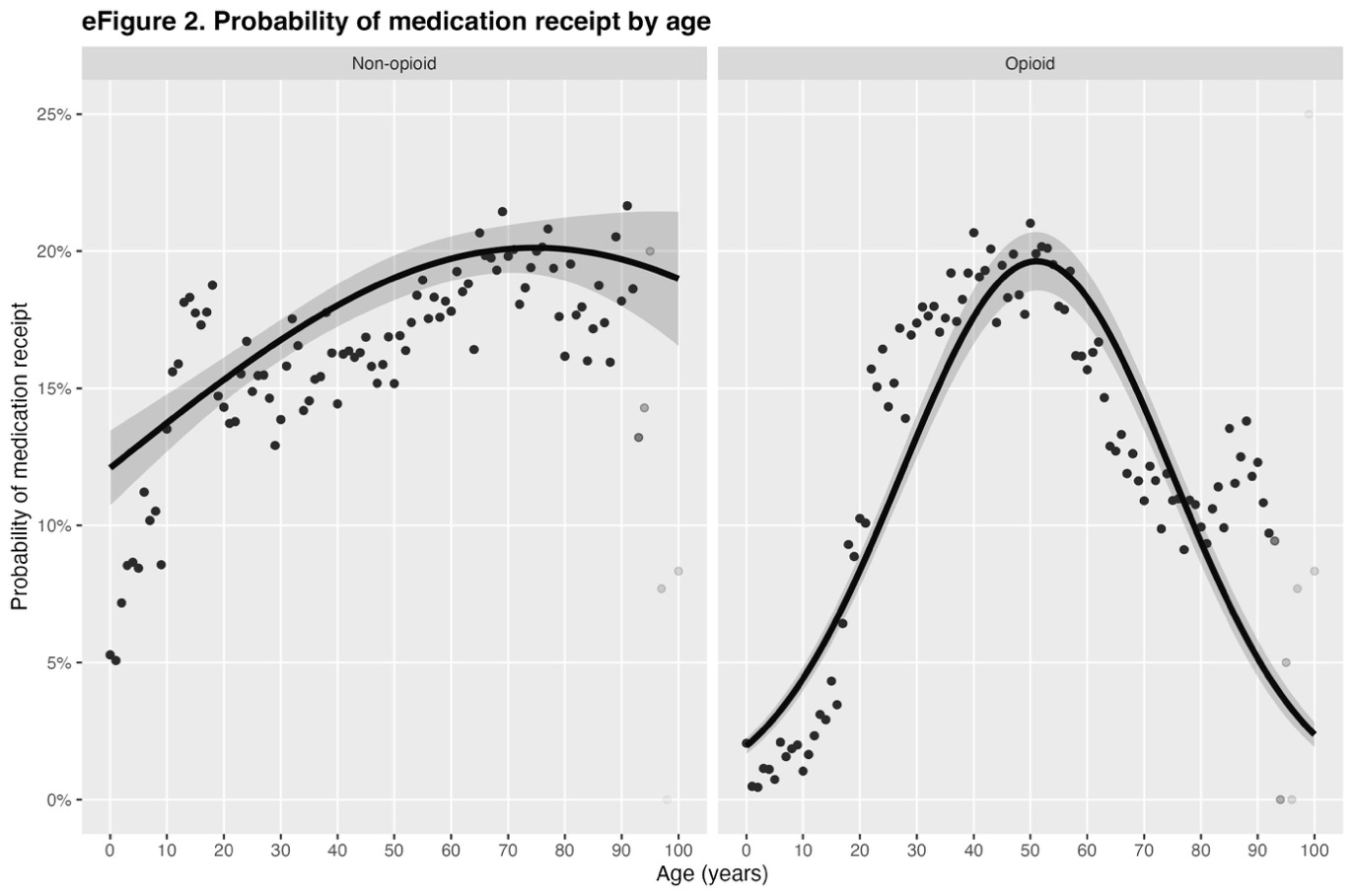


## eFigure 3. Most common opioid medications provided for pain-related visits.

## eFigure 4. Most common non-opioid analgesics for pain-related visits when no opioids provided.

# Appendices

## Appendix A. STROBE Checklist.

STROBE Statement—Checklist of items that should be included in reports of ***cross-sectional studies***

|  | Item No | Recommendation | Page No |
| --- | --- | --- | --- |
| **Title and abstract** | 1 | (*a*) Indicate the study’s design with a commonly used term in the title or the abstract | 1 |
|  |  | (*b*) Provide in the abstract an informative and balanced summary of what was done and what was found | 2 |
| Introduction | | | |
| Background/rationale | 2 | Explain the scientific background and rationale for the investigation being reported | 3-4 |
| Objectives | 3 | State specific objectives, including any prespecified hypotheses | 4 |
| Methods | | | |
| Study design | 4 | Present key elements of study design early in the paper | 4-5 |
| Setting | 5 | Describe the setting, locations, and relevant dates, including periods of recruitment, exposure, follow-up, and data collection | 4-5 |
| Participants | 6 | (*a*) Give the eligibility criteria, and the sources and methods of selection of participants | 4,6 |
| Variables | 7 | Clearly define all outcomes, exposures, predictors, potential confounders, and effect modifiers. Give diagnostic criteria, if applicable | 5-7 |
| Data sources/ measurement | 8* | For each variable of interest, give sources of data and details of methods of assessment (measurement). Describe comparability of assessment methods if there is more than one group | 5-7 |
| Bias | 9 | Describe any efforts to address potential sources of bias | 8-9 |
| Study size | 10 | Explain how the study size was arrived at | 5 |
| Quantitative variables | 11 | Explain how quantitative variables were handled in the analyses. If applicable, describe which groupings were chosen and why | 5-7 |
| Statistical methods | 12 | (*a*) Describe all statistical methods, including those used to control for confounding | 7-8 |
|  |  | (*b*) Describe any methods used to examine subgroups and interactions | 7-8 |
|  |  | (*c*) Explain how missing data were addressed | 6,8 |
|  |  | (*d*) If applicable, describe analytical methods taking account of sampling strategy | 8 |
|  |  | (*e*) Describe any sensitivity analyses | 8-9 |
| Results | | | |
| Participants | 13* | (a) Report numbers of individuals at each stage of study—eg numbers potentially eligible, examined for eligibility, confirmed eligible, included in the study, completing follow-up, and analysed | 9-10 |
|  |  | (b) Give reasons for non-participation at each stage | na |
|  |  | (c) Consider use of a flow diagram | na |
| Descriptive data | 14* | (a) Give characteristics of study participants (eg demographic, clinical, social) and information on exposures and potential confounders | 9-10 |
|  |  | (b) Indicate number of participants with missing data for each variable of interest | 13 |
| Outcome data | 15* | Report numbers of outcome events or summary measures | Table |
| Main results | 16 | (*a*) Give unadjusted estimates and, if applicable, confounder-adjusted estimates and their precision (eg, 95% confidence interval). Make clear which confounders were adjusted for and why they were included | 10-12 |
|  |  | (*b*) Report category boundaries when continuous variables were categorized | na |
|  |  | (*c*) If relevant, consider translating estimates of relative risk into absolute risk for a meaningful time period | 10,11 |
| Other analyses | 17 | Report other analyses done—eg analyses of subgroups and interactions, and sensitivity analyses | 13 |
| Discussion | | | |
| Key results | 18 | Summarise key results with reference to study objectives | 13,14 |
| Limitations | 19 | Discuss limitations of the study, taking into account sources of potential bias or imprecision. Discuss both direction and magnitude of any potential bias | 16,17 |
| Interpretation | 20 | Give a cautious overall interpretation of results considering objectives, limitations, multiplicity of analyses, results from similar studies, and other relevant evidence | 17 |
| Generalisability | 21 | Discuss the generalisability (external validity) of the study results | 14 |
| Other information | | | |
| Funding | 22 | Give the source of funding and the role of the funders for the present study and, if applicable, for the original study on which the present article is based | 17 |

*Give information separately for exposed and unexposed groups.

**Note:** An Explanation and Elaboration article discusses each checklist item and gives methodological background and published examples of transparent reporting. The STROBE checklist is best used in conjunction with this article (freely available on the Web sites of PLoS Medicine at http://www.plosmedicine.org/, Annals of Internal Medicine at http://www.annals.org/, and Epidemiology at http://www.epidem.com/). Information on the STROBE Initiative is available at www.strobe-statement.org.

## Appendix B. Classification of pain-related visits

We examined all National Center for Health Statistics (NCHS) reason for visit classification (RVC) codes and identified 53 relating to a pain-based symptom or condition and categorized these *a priori* under 8 broad classes of pain as described below. The original list of RVC codes were published in 1979^1^ with any annual changes to the original classification codes listed in NAMCS documentation^2^. Although there were some documented changes to classification codes across survey years, we could find none that related to classifications of interest to the current study.

| **Pain class** | **Pain type** |
| --- | --- |
| Musculoskeletal pain | symptoms of pain, ache, soreness, discomfort for: 1900.1 Neck, 1905.1 Back, 1910.1 Low back, 1915.1 Hip, 1920.1 Leg, 1925.1 Knee, 1930.1 Ankle, 1935.1 Foot and toe, 1940.1 Shoulder, 1945.1 Arm, 1950.1 Elbow, 1955.1 Wrist, 1960.1 Hand and finger, 1965.1 unspecified muscles, 1970.1 unspecified joints, 1980.1 Other musculoskeletal symptoms (Includes bone pain, stump pain) |
| Abdominal pain | 1545.0 stomach and abdominal pain, cramps and spasms, 1545.1 abdominal pain, cramps, spasms, NOS, 1545.2 lower abdominal pain, cramps, spasms, 1545.3 upper abdominal pain, cramps, spasms |
| Chest Pain | 1050.1 Chest pain (excludes heart pain) |
| Headache | 1210.0 Headache, pain in head |
| Ear/eye pain | 1320.1 Eye pain, 1355.1 Earache - pain |
| Dental pain | 1500.1 toothache, 1500.2 gum pain, 1510.1 mouth - pain, burning, soreness |
| Genitourinary pain | 1650.0 painful urination, 1670.1 bladder pain, 1670.1 kidney pain, 1700.1 symptoms of penis - pain, aching, soreness, 1715.1 symptoms of the scrotum and testes - pain, aching, tenderness, 1745.2 painful menstruation (dysmenorrhea), 1765.1 vaginal symptoms - pain, 1775.1 pelvic symptoms – pain, 1790.1 pain during pregnancy, 1790.3 symptoms of onset of labor, including labor pain, 1791.0 postpartum pain, 1800.0 Pain or soreness of breast |
| General/other pain | 1055.0 pain, specified site not referable to a specific body system (inc. buttock pain, gluteal pain), 1055.1 rib pain, 1055.2 side pain, 1055.3 groin pain, 1055.4 facial pain, 1060.1 pain generalized, site unspecified, 1870.1 skin pain, 14552 throat pain, 1458.1 lung pain, 1515.1 symptoms referable to tongue - pain, 1605.1 symptoms referable to anus-rectum – pain, 1610.1 symptoms of liver, gallbladder, and biliary tract – pain, 4205.0 post operative visit (includes pain), 2675.5 TMJ pain |

## Appendix C. Combining annual data across survey years

We followed NCHS trend analysis documentation^3^ for combining data across different survey years to account for alterations in survey content or coding and this is summarized below (there were some minor changes to variable names or value labels which we accounted for but do not document here).

*Payment type*. The NAMCS patient record provides seven different options for expected source of payment. Prior to 2005 only one of these options was selectable as the main source of payment, while from 2005 onwards multiple options could be selected. Following NAMCS guidance, we recoded the ≥2005 year multi-response options to a single *main* expected source of payment based on a pre-defined NAMCS hierarchy^4^ to ensure consistency with pre-2005 coding.

*Medication*. Since 1991, NAMCS has classified medications using the proprietary NCHS system, with the Multum Lexicon added as an additional categorization system in 2006. While the Multum system provides a single code for all variations of the same generic ingredient (e.g., Ibuprofen), multiple NCHS codes are assigned for brand variations (e.g., Advil, Motrin). NAMCS provides SAS files^3^ to convert NCHS codes to the corresponding Multum codes allowing data to be easily combined across survey years, which we adapted for use in R. We also used NAMCS-provided SAS scripts to create cluster weights for the 1991-2001 survey years as these are not explicitly provided in the dataset (although data variables to allow their computation is provided). While medication data are collected across all survey years, the number of recorded medications increased from 6 (in 1999) to 30 (in 2020). As recommended, we extracted the first 6 medications for all survey years for consistency.

Data were not collected in select survey years for Metropolitan Status Area (2012) or census region (2018-2019), and there were missing data for pain chronicity (N = 1,614) and new patient status (N = 129) and so these missing data could not be included in covariate analysis.

## Appendix D. NAMCS patient record form instructions for coding ethnicity and race

**Ethnicity**

Ethnicity refers to a person's national or cultural group.^2^ There are two categories for ethnicity, "Hispanic or Latino" and "Not Hispanic or Latino". Enter the appropriate category according to the information in the medical record. If the patient's ethnicity is not known and is not obvious, enter the category which in your judgment is most appropriate. The definitions of the categories are listed below. Do not determine the patient’s ethnicity from his/her last name.

1 - Hispanic or Latino

*A person of Cuban, Mexican, Puerto Rican, South or Central American or other Spanish culture or origin regardless of race.*

2- Not Hispanic or Latino

*All other persons.*

**Race**

Enter all appropriate categories based on the information in medical record. If the patient's race is not known or not obvious, enter the categories which in your judgment is (are) most appropriate. Do not determine the patient's race from their last name.

1-White.

*A person having origins in any of the original peoples of Europe, the Middle East, or North Africa.*2-Black or African American.

*A person having origins in any of the black racial groups of Africa.*

3 – Asian.

*A person having origins in any of the original peoples of the Far East, Southeast Asia, or the Indian subcontinent including, for example, Cambodia, China, India, Japan, Korea, Malaysia, Pakistan, the Philippine Islands, Thailand, and Vietnam.*

4-Native Hawaiian or Other Pacific Islander.

*A person having origins in any of the original peoples of Hawaii, Guam, Samoa, or other Pacific Islands.*5-American Indian or Alaska Native.

*A person having origins in any of the original peoples of North America, and who maintains cultural identification through tribal affiliation or community recognition.*

## Appendix E. ICD-CM classification codes for alcohol or substance abuse/dependence

The following ICD-9-CM^5^ (1999-2015 surveys) and ICD-10-CM^6^ (2016-2020) diagnostic codes were used to classify alcohol or substance abuse/dependence

**Alcohol abuse/dependence**

*ICD-9-CM*

291 Alcohol-induced mental disorders

303 Alcohol dependence syndrome

305.0 Nondependent alcohol abuse

*ICD-10-CM*

F10 Alcohol related disorders

**Substance abuse/dependence**

*ICD-9-CM*

[Drug-induced mental disorders 292]

292 (drug induced disorder, intoxication or withdrawal)

[Drug dependence 304]

304.0 Opioid type dependence

304.1 Sedative, hypnotic or anxiolytic dependence

304.2 Cocaine dependence

304.3 Cannabis dependence

304.4 Amphetamine and other psychostimulant dependence

304.5 Hallucinogen dependence

304.6 Other specified drug dependence

[Nondependent abuse of drugs 305]

305.2 Nondependent cannabis abuse

305.3 Nondependent hallucinogen abuse

305.4 Nondependent sedative, hypnotic or anxiolytic abuse

305.5 Nondependent opioid abuse

305.6 Nondependent cocaine abuse

305.7 Nondependent amphetamine or related acting sympathomimetic abuse

*ICD-10-CM*

[Chapter 5: Mental and behavioral disorders due to psychoactive substance use (F10-F19)]

F11 Opioid related disorders

F12 Cannabis related disorders

F13 Sedative, hypnotic, or anxiolytic related disorders

F14 Cocaine related disorders

F15 Other stimulant related disorders

F16 Hallucinogen related disorders

F18 Inhalant related disorders

F19 Other psychoactive substance related disorders

# References

1. Schneider D, Appleton L, McLemore T. A reason for visit classification for ambulatory care. *Vital Health Stat 2*. 1979i-vi, 1.

2. National Center For Health Statistics. NAMCS documentation. Published 2023. Accessed 4 Jun, 2023. https://ftp.cdc.gov/pub/Health_Statistics/NCHS/Dataset_Documentation/NAMCS/

3. National Center For Health Statistics. Trend Analysis Using NAMCS and NHAMCS Drug Data. Published 2021. Accessed 4 June, 2023. https://www.cdc.gov/nchs/ahcd/trend_analysis.htm

4. National Center For Health Statistics. Notices for NAMCS and NHAMCS Public Use Data File Users. Centers for Disease Control and Prevention. Published 2023. Accessed 17 May, 2023. https://www.cdc.gov/nchs/ahcd/notice.htm

5. National Center For Health Statistics. International Classification of Diseases,Ninth Revision, Clinical Modification (ICD-9-CM). Centers For Disease Control And Prevention. Published 2021. Accessed 5 May, 2023. https://www.cdc.gov/nchs/icd/icd9cm.htm

6. National Center For Health Statistics. International Classification of Diseases, Tenth Revision, Clinical Modification. Centers For Disease Control And Prevention. Published 2022. Accessed 5 May, 2023. https://www.cdc.gov/nchs/icd/icd-10-cm.htm
