# Supplementary material for: Racial and Ethnic Disparities in the Prescribing of Pain Medication in US Primary Care Settings, 1999–2019: Where Are We Now?
Source: J Gen Intern Med. 2024 Feb 1;39(9):1597–605. doi: 10.1007/s11606-024-08638-5 (PMC11254888; doi:10.1007/s11606-024-08638-5)
Supplement: Supplementary file 2 — Supplementary file2 (DOCX 33 KB) [file 11606_2024_8638_MOESM2_ESM.docx]

Table S1. Population-weighted odds ratios [95% Confidence Intervals] of receiving medication during pain-related visits to office-based physicians (N=77,748) derived from multinomial regression.

| **Variable** |  |  |  |  |  |  |
| --- | --- | --- | --- | --- | --- | --- |
| **Race** | **Reference** | **Outcome**  **(vs. no medicaton reference)** | **Odds Ratio** | **Lower 95% CI** | **Upper**  **95% CI** | **p** |
| Black | White | Opioids | 0.99 | 0.86 | 1.14 | 0.873 |
| Black |  | Non-opioids only | 1.19 | 1.07 | 1.31 | <.001 |
| Black |  | Opioids + non-opioids | 1.08 | 0.89 | 1.31 | 0.418 |
| Other |  | Opioids | 0.53 | 0.38 | 0.73 | <.001 |
| Other |  | Non-opioids only | 0.93 | 0.68 | 1.27 | 0.647 |
| Other |  | Opioids + non-opioids | 0.59 | 0.39 | 0.88 | 0.010 |
| **Ethnicity** |  |  |  |  |  |  |
| Hispanic or Latino | Not Hispanic/Latino | Opioids | 0.74 | 0.64 | 0.86 | <.001 |
| Hispanic or Latino |  | Non-opioids only | 1.28 | 1.16 | 1.43 | <.001 |
| Hispanic or Latino |  | Opioids + non-opioids | 0.82 | 0.68 | 0.99 | 0.043 |
| **Sex** |  |  |  |  |  |  |
| Male | Female | Opioids | 1.09 | 1.01 | 1.18 | 0.027 |
| Male |  | Non-opioids only | 1.07 | 1.01 | 1.14 | 0.019 |
| Male |  | Opioids + non-opioids | 1.15 | 1.02 | 1.29 | 0.025 |
| **Insurance** |  |  |  |  |  |  |
| Medicaid | Private | Opioids | 1.68 | 1.46 | 1.94 | <.001 |
| Medicaid |  | Non-opioids only | 1.12 | 0.99 | 1.26 | 0.074 |
| Medicaid |  | Opioids + non-opioids | 1.37 | 1.11 | 1.7 | 0.004 |
| Medicare |  | Opioids | 1.59 | 1.41 | 1.8 | <.001 |
| Medicare |  | Non-opioids only | 1.03 | 0.94 | 1.14 | 0.498 |
| Medicare |  | Opioids + non-opioids | 1.33 | 1.10 | 1.61 | 0.004 |
| No insurance |  | Opioids | 1.49 | 1.18 | 1.89 | <.001 |
| No insurance |  | Non-opioids only | 0.88 | 0.73 | 1.07 | 0.193 |
| No insurance |  | Opioids + non-opioids | 1.04 | 0.76 | 1.44 | 0.792 |
| Unknown |  | Opioids | 1.21 | 1.01 | 1.46 | 0.042 |
| Unknown |  | Non-opioids only | 0.97 | 0.84 | 1.12 | 0.689 |
| Unknown |  | Opioids + non-opioids | 1.17 | 0.91 | 1.51 | 0.223 |
| **Alcohol Disorder** |  |  |  |  |  |  |
| Yes | No | Opioids | 1.59 | 1.28 | 1.99 | <.001 |
| Yes |  | Non-opioids only | 1.32 | 1.04 | 1.67 | 0.021 |
| Yes |  | Opioids + non-opioids | 1.95 | 1.44 | 2.64 | <.001 |
| **Substance Disorder** |  |  |  |  |  |  |
| Yes | No | Opioids | 4.21 | 2.72 | 6.51 | <.001 |
| Yes |  | Non-opioids only | 1.69 | 1.09 | 2.62 | 0.018 |
| Yes |  | Opioids + non-opioids | 4.37 | 2.08 | 9.19 | <.001 |
| **Pain chronicity** |  |  |  |  |  |  |
| Chronic | Acute | Opioids | 1.49 | 1.35 | 1.66 | <.001 |
| Chronic |  | Non-opioids only | 0.71 | 0.66 | 0.77 | <.001 |
| Chronic |  | Opioids + non-opioids | 1.03 | 0.9 | 1.18 | 0.668 |
| Pre/post Surgery |  | Opioids | 0.73 | 0.63 | 0.84 | <.001 |
| Pre/post Surgery |  | Non-opioids only | 0.44 | 0.38 | 0.5 | <.001 |
| Pre/post Surgery |  | Opioids + non-opioids | 0.57 | 0.44 | 0.74 | <.001 |
| Preventive care |  | Opioids | 0.54 | 0.29 | 0.99 | 0.04~8 |
| Preventive care |  | Non-opioids only | 0.6 | 0.46 | 0.77 | <.001 |
| Preventive care |  | Opioids + non-opioids | 0.46 | 0.26 | 0.79 | 0.005 |
| **Pain Class** |  |  |  |  |  |  |
| Musculoskeletal | Abdominal | Opioids | 2.67 | 2.25 | 3.18 | <.001 |
| Musculoskeletal |  | Non-opioids only | 3.92 | 3.34 | 4.59 | <.001 |
| Musculoskeletal |  | Opioids + non-opioids | 7 | 4.24 | 11.55 | <.001 |
| Other |  | Opioids | 1.78 | 1.47 | 2.15 | <.001 |
| Other |  | Non-opioids only | 1.82 | 1.52 | 2.17 | <.001 |
| Other |  | Opioids + non-opioids | 2.69 | 1.6 | 4.53 | <.001 |
| Eye/ear |  | Opioids | 0.63 | 0.49 | 0.82 | <.001 |
| Eye/ear |  | Non-opioids only | 0.98 | 0.81 | 1.18 | 0.800 |
| Eye/ear |  | Opioids + non-opioids | 0.9 | 0.45 | 1.79 | 0.768 |
| Headache |  | Opioids | 1.66 | 1.33 | 2.07 | <.001 |
| Headache |  | Non-opioids only | 2.68 | 2.23 | 3.22 | <.001 |
| Headache |  | Opioids + non-opioids | 2.72 | 1.6 | 4.62 | <.001 |
| Chest |  | Opioids | 0.9 | 0.65 | 1.25 | 0.541 |
| Chest |  | Non-opioids only | 3.26 | 2.72 | 3.9 | <.001 |
| Chest |  | Opioids + non-opioids | 2.31 | 1.24 | 4.33 | 0.008 |
| Genitourinary |  | Opioids | 0.7 | 0.53 | 0.92 | 0.012 |
| Genitourinary |  | Non-opioids only | 1 | 0.81 | 1.23 | 0.986 |
| Genitourinary |  | Opioids + non-opioids | 0.73 | 0.37 | 1.43 | 0.359 |
| Dental |  | Opioids | 11.85 | 6.85 | 20.5 | <.001 |
| Dental |  | Non-opioids only | 3.1 | 1.74 | 5.53 | <.001 |
| Dental |  | Opioids + non-opioids | 8.18 | 3.41 | 19.62 | <.001 |
| **Patient status** |  |  |  |  |  |  |
| New patient | Existing | Opioids | 0.6 | 0.53 | 0.68 | <.001 |
| New patient |  | Non-opioids only | 0.83 | 0.76 | 0.9 | <.001 |
| New patient |  | Opioids + non-opioids | 0.75 | 0.64 | 0.88 | <.001 |
| **Region** |  |  |  |  |  |  |
| Midwest | Northeast | Opioids | 1.28 | 1.06 | 1.55 | 0.010 |
| Midwest |  | Non-opioids only | 1.11 | 0.96 | 1.28 | 0.160 |
| Midwest |  | Opioids + non-opioids | 1.25 | 0.98 | 1.58 | 0.066 |
| South |  | Opioids | 1.53 | 1.28 | 1.83 | <.001 |
| South |  | Non-opioids only | 1.05 | 0.93 | 1.19 | 0.425 |
| South |  | Opioids + non-opioids | 1.39 | 1.1 | 1.76 | 0.005 |
| West |  | Opioids | 1.44 | 1.19 | 1.74 | <.001 |
| West |  | Non-opioids only | 1.07 | 0.92 | 1.24 | 0.356 |
| West |  | Opioids + non-opioids | 1.62 | 1.26 | 2.08 | 1e-04 |
| **Metropolitan Area** |  |  |  |  |  |  |
| Urban | Rural | Opioids | 0.85 | 0.7 | 1.04 | 0.1081 |
| Urban |  | Non-opioids only | 0.95 | 0.83 | 1.09 | 0.4633 |
| Urban |  | Opioids + non-opioids | 0.98 | 0.76 | 1.25 | 0.8467 |
| **Age** |  |  |  |  |  |  |
| Linear |  | Opioids | 1.2 | 1.12 | 1.29 | <.001 |
| Linear |  | Non-opioids only | 1.19 | 1.13 | 1.24 | <.001 |
| Linear |  | Opioids + non-opioids | 1.28 | 1.16 | 1.42 | <.001 |
| Quadratic |  | Opioids | 0.53 | 0.5 | 0.56 | <.001 |
| Quadratic |  | Non-opioids only | 0.9 | 0.87 | 0.94 | <.001 |
| Quadratic |  | Opioids + non-opioids | 0.55 | 0.49 | 0.61 | <.001 |
| **Year** |  |  |  |  |  |  |
| Linear |  | Opioids | 1.3 | 1.23 | 1.38 | <.001 |
| Linear |  | Non-opioids only | 1.1 | 1.04 | 1.15 | <.001 |
| Linear |  | Opioids + non-opioids | 1.33 | 1.23 | 1.44 | <.001 |
| Quadratic |  | Opioids | 0.95 | 0.89 | 1.01 | 0.096 |
| Quadratic |  | Non-opioids only | 1.08 | 1.02 | 1.14 | 0.004 |
| Quadratic |  | Opioids + non-opioids | 1.06 | 0.98 | 1.15 | 0.174 |
